# Flexural Strength and Hardness Analysis of 3D-Printed vs. Milled Resin Composites Indicated for Definitive Crowns

**DOI:** 10.3390/jfb16120468

**Published:** 2025-12-18

**Authors:** Hunaida Khaled Tayeb, Nick Silikas, Abdulrahman Jafar Alhaddad, Julian Satterthwaite

**Affiliations:** 1 Oral and Maxillofacial Prosthodontics Department, Faculty of Dentistry, King Abdulaziz University, Jeddah 21589, Saudi Arabia; aalhaddad@kau.edu.sa; 2 Division of Dentistry, School of Medical Sciences, University of Manchester, Manchester M13 9PL, UK; nikolaos.silikas@manchester.ac.uk (N.S.); julian.satterthwaite@manchester.ac.uk (J.S.)

**Keywords:** 3D-printing, CAD/CAM, crowns, Martens hardness, flexural strength, hardness

## Abstract

The growing use of 3D-printed dental restorations has created a need to understand how the mechanical behaviour of definitive 3D-printed resin composites compares with milled counterparts. This study compared the mechanical properties of 3D-printed and milled definitive crowns and examined the factors affecting these properties. The 3D-printed materials (Permanent Crown Resin: PCR, VarseoSmile Crown Plus: VCP, and Crowntec: CT) and milled blocks (Brilliant Crios: BC, Shofu Block HC: HC, and Grandio Blocs: Gr) were tested. Filler content was evaluated using the ash method (*n* = 3), and flexural strength (FS) and flexural modulus (E*_f_*) were assessed using a three-point bending test (*n* = 10). Martens hardness (HM), indentation modulus, and Vickers hardness were determined using the Martens indentation test (*n* = 24). Vickers hardness was also measured with the Vickers indenter tester (*n* = 24). Statistical analysis investigated differences between materials and methods, and correlations between filler weight and mechanical properties (α = 0.05). FS of milled blocks, Gr (244.5 MPa), BC (225.5 MPa), and HC (155 MPa), were higher than that of the 3D-printed resin composites: PCR (143.6 MPa), CT (140.9 MPa), and VCP (128 MPa). Measured mechanical properties of the milled blocks were significantly higher than those of the 3D-printed materials (*p* < 0.001). HM of the 3D-printed materials was similar (≈217 N/mm^2^), while HM of the milled blocks ranged from 434.7 to 858.4 N/mm^2^. The 3D-printed materials showed comparable properties; however, milled blocks differed significantly (*p* < 0.05). Filler content was strongly associated with FS and HM. Within the limitations of this study, the milled resin composites showed markedly higher strength and hardness, while 3D-printed materials may be suitable for low-to-moderate load clinical scenarios. Further studies to evaluate the long-term performance of the 3D-printed resin composites are recommended.

## 1. Introduction

Dental restorations have evolved significantly over the past few decades, with the development of new materials and fabrication technologies that have improved the quality and efficiency of dental treatments. Among the many innovations, computer-aided design and computer-aided manufacturing (CAD/CAM) technology have revolutionised the fabrication of dental prostheses, allowing for the production of highly accurate and customised restorations [1] and have led to improved clinical results, patient satisfaction [2], and reduced chairside time [3]. More recently, 3D printing has emerged as another promising technology in dentistry, offering the potential for rapid prototyping and production of complex dental restorations with high precision, efficiency, and patient customisation [2].

Dental crowns play an essential role in dentistry to restore form, function, and aesthetics to teeth [2,4]. Traditionally, dental crowns have been fabricated using various materials, such as metal, porcelain fused to metal, and all-ceramic, each with advantages and disadvantages [5]. With the advent of CAD/CAM subtractive manufacturing, the production of dental crowns has become more efficient and precise, enabling the use of materials with superior mechanical properties and aesthetics, such as zirconia and lithium disilicate [2]. This method uses milling machines to fabricate restorations from solid blocks of dental materials [6].

Three-dimensional printing, also known as additive manufacturing, has gained increasing attention in the dental field due to its potential to overcome some limitations of other manufacturing methods, including milling. This technology allows for the creation of dental restorations by depositing successive layers of material, enabling the fabrication of complex shapes and geometries that may be difficult or impossible to achieve through milling [7]. Additionally, 3D printing offers the potential for reduced material waste, shorter production times, and lower costs when compared with CAD/CAM milling [8].

The mechanical properties of 3D-printed definitive restorations are crucial to ensure their long-term success and durability in clinical applications. Flexural strength is an essential measure of the ability of a material to resist deformation and fracture under bending loads [9]. Hardness is a crucial property that influences the wear resistance, abrasion resistance, and overall durability of dental materials [10]. Martens hardness and Vickers hardness are two standard methods used to evaluate the hardness of dental materials, providing valuable information about their performance and potential longevity in clinical applications [11].

Several studies have investigated the mechanical properties of milled dental restorations, demonstrating their suitability for various clinical applications [12]. In contrast, existing research on 3D-printed materials remains comparatively limited, with most studies focusing on provisional resin composites or evaluating a single printing technology [13,14]. Differences among 3D printing technologies, such as SLA and DLP systems, which employ distinct curing mechanisms, may contribute to variations in mechanical behaviour; however, these effects remain insufficiently characterised. Furthermore, the consistency of hardness measurements obtained using different indentation testers has not yet been fully investigated for 3D-printed and milled resin composites.

A direct formulation-matched comparison between additive and subtractive manufacturing is not yet feasible, as no identical resin composites are commercially available in both forms. Therefore, the present study assessed commercially available representative materials from each fabrication technique to provide clinically relevant insight into performance differences across technologies. Accordingly, this study aimed to provide a multi-material, cross-technology comparison of the mechanical properties of 3D-printed and milled resin composites indicated for definitive crowns, focusing on the flexural strength (FS), flexural modulus (E*_f_*), Martens hardness (HM), indentation modulus (E*_iT_*), and Vickers hardness (HV) of these restorations.

The null hypotheses were as follows:

1. There is no significant difference in the mechanical properties of the investigated materials between the 3D-printed and milled resin composites indicated for definitive crowns.
2. The composition of the evaluated materials does not affect their mechanical properties.

## 2. Materials and Methods

### 2.1. Materials

Six commercially available resin composites for digitally fabricated definitive crowns were tested. There were three 3D-printed materials, Permanent Crown Resin (PCR), VarseoSmile Crown Plus (VCP), and Crowntec (CT), and three milled blocks, Brilliant Crios (BC), Shofu Block HC (HC), and Grandio Blocs (Gr) (Table 1).

### 2.2. Specimen Preparation

**Figure 1:**
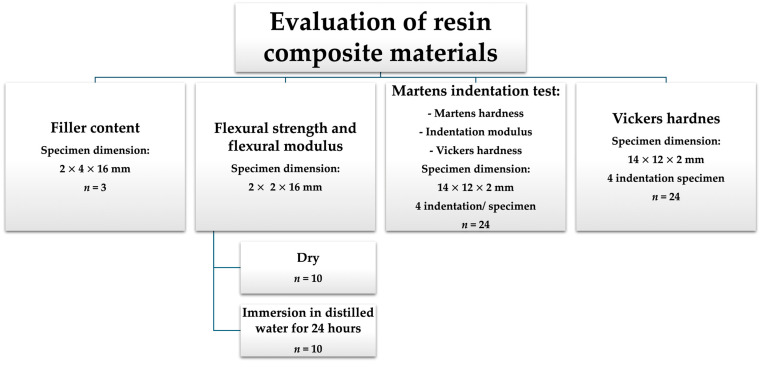
Study design.

Specimen dimensions varied according to each experiment (Figure 1), and they were verified to an accuracy of ±0.01 mm, using a digital micrometre (Digimatic Micrometers, QuantuMike IP65, Mitutoyo, Japan). Any specimen not within this range was rejected and discarded. Although the investigated materials are indicated for definitive crowns, all tests were performed on standardised shaped specimens following ISO 1172, 4049, and 14,577 requirements. All tests were conducted under controlled laboratory conditions (23 ± 1 °C and 50 ± 10% relative humidity), in accordance with ISO recommendations.

The sample sizes (Figure 1) for filler content and flexural tests followed a previous validated study [13]. For the Martens indentation and Vickers hardness tests, sample sizes were determined using G*power software (Version 3.1.3; Heinrich Heine University, Germany), where an effect size (f = 0.40) was used, corresponding to a moderate-to-large effect and selected to reflect the substantial differences typically observed among resin composites, at a significance level of α = 0.05.

#### 2.2.1. Milled Resin Composite Blocks

Specimens of each milled resin composite block were securely mounted to a low-speed water-cooled diamond saw (Isomet 1000 Precision Saw; Buehler Co., Lake Bluff, IL, USA) and sectioned under constant water irrigation.

#### 2.2.2. 3D-Printed Resin Composites

Specimens of the 3D-printed materials were designed as standard tessellation language (STL) files using an online 3D-modelling programme (Tinkercad software, an online 3D-modelling programme). The STL file was exported to a 3D-printer slicing and preparation software, Preform version 3.23.1 (Formlabs, Somerville, MA, USA), for PCR, and Asiga Composer version 1.3.5 (Asiga, Sydney, Australia) for VCP and CT. Specimens were aligned to be printed in a vertical orientation. The materials were then printed at a 50 µm layer thickness using compatible 3D printers; PCR was printed using Form 3B (Formlabs, Somerville, MA, USA), and CT and VCP were printed using Asiga MAX (Asiga, Sydney, Australia). The printed specimens were then ultrasonically cleaned in Form Wash (Formlabs, Somerville, MA, USA), with either 99% isopropyl alcohol (PCR and CT) and ethanol 96% (VCP) for 3 min, then air dried and post-cured according to the manufacturer’s directions (Table 2). Support structures were removed from the printed specimens, and the surface was finished and polished.

### 2.3. Evaluation of Filler Content

Specimens of the investigated materials (*n* = 3 of each material) with dimensions of (2 × 4 × 16 mm) were fabricated. Each sample was heated at a constant temperature (ash method) following ISO standard 1172:1996 [15]. Samples were heated at 600 °C for 30 min using an electric furnace (Programat EP 5000, Ivoclar Vivadent, Schaan, Liechtenstein). The samples were weighed before and after heating to an accuracy of 0.01 mg using a calibrated electronic analytical balance.

The filler weight percentage was calculated using the following formula:

Filler weight % = (m_2_/m_1_) × 100

where m_1_ is the mass before heating and m_2_ is the mass after heating and cooling.

### 2.4. Evaluation of the Flexural Strength

A total of 20 bar-shaped specimens of each investigated material were fabricated with dimensions of 2 × 2 × 16 mm. The prominent edges of each specimen were manually smoothed using a 1200-grit abrasive disc. Prepared specimens were randomly allocated into two groups (*n* = 10). The first group, which served as the baseline, was stored in dry conditions and at room temperature for 24 ± 2 h; the second group was immersed in distilled water at 37 °C for 24 ± 2 h. This storage duration follows the ISO 4049 requirement for baseline flexural strength testing [16].

To perform a three-point bending test, specimens were placed in a universal testing machine with a 0.5-kN cell load on 12 mm separated supports and loaded to failure from a point source at the top centre of the bar at a crosshead speed of 1 mm/min. Flexural strength (σ) and modulus (E*_f_*) calculations were based on the following formulas, as in ISO 4049:2019 [16]:

$$\sigma =\;\frac{3FL}{2bh^{2}}$$

$$E_{f}=\frac{L^{3}F}{4bh^{3}d}$$

where F is the maximum applied load, in newtons; L is the distance, in millimetres, between the supports, i.e., 12 mm; b is the width of the test specimen, in millimetres; h is the height of the test specimen, in millimetres; and d is the deflection in millimetres.

### 2.5. Evaluation of the Hardness

Additional specimens were prepared for each hardness evaluation method. In accordance with ISO 14577-4:2016 [17] guidelines for Martens hardness measurements, the specimens were finished and polished by a single operator to ensure consistency. Surface roughness was confirmed to be below 0.2 μm (Ra < 0.2 μm), using a non-contact profilometer (Talysurf CLI 1000, Taylor Hobson Precision, Leicester, UK). The polishing process was performed on the upper surface of each specimen, utilising silicon carbide paper in sequential grits of 600, 800, 1000, and 1200 (P2500) at 300 rpm under water cooling (60 s) using MetaServ 250 Grinder-Polishers (Buehler, IL, USA). To check the parallelism, the upper surface of each specimen was visually inspected and then checked using a digital micrometre to ensure dimensions were within ±0.1 mm.

#### 2.5.1. Martens Hardness

Six specimens of each material were fabricated with dimensions of 14 × 12 × 2 mm. Four force-controlled indentations (*n* = 24 for each material) were made on the polished surface of each specimen using a Zwick Martens Hardness Instrument (Z2.5, ZwickRoell Ltd., Leominster, UK) with a Vickers hardness measuring tip. A force of up to 10 N was applied at a loading speed of 5 N/s, maintained for 5 s, and then removed at a rate of 5 N/s. The Zwick TestXpert^®^ programme automatically calculated Martens hardness (HM) in (N/mm^2^), indentation modulus (E*_IT_*) in (kN/mm^2^), and Vickers hardness (HV).

#### 2.5.2. Vickers Hardness

Six specimens of each material were fabricated with dimensions of 16 × 4 × 2 mm. A Vickers indenter tester (FM-700; Future Tech Corp., Japan) was used to measure the microhardness of each material under a load of 300 g for a 20 s dwell time. A microscope at ×50 magnification measured each indentation diagonal (D1, D2). Then, the machine automatically calculated the corresponding hardness value and presented it as a Vickers hardness number (VHN). Vickers hardness number (VHN) can also be calculated using the following formula [18]:

$$\mathrm{VHN}\;=\;1.854\;\times \;\frac{P}{D^{2}}$$

where P is the applied load in kg, and D is the indentation diagonal length in mm.

The hardness values were averaged after making four indents on the polished surface of each sample (*n* = 24 for each material) in a straight line 0.5 mm from the sample edges. To guarantee appropriate spacing between the indentations, the average indentation diagonal length was multiplied by four (4 × D) to obtain the distance between the indentations.

### 2.6. Statistical Analysis

The data were analysed using statistical software (IBM SPSS Statistics, Version 28; IBM Corp). Means and standard deviations (SD) were obtained. The normal distribution of the data was checked using the Shapiro–Wilk test. The homogeneity of variance was checked using Levene’s test. All statistical tests were performed at a significance level of α = 0.05.

Flexural strength (FS) and flexural modulus (E*_f_*) were analysed using a two-way ANOVA with material and storage conditions specified as fixed factors. For the other properties (Martens hardness, indentation modulus, and Vickers hardness), one-way ANOVA followed by Bonferroni post hoc tests was used to assess differences among materials. An independent samples *t*-test was performed to compare flexural and indentation modulus and Vickers hardness data of the investigated materials (measured by Zwick vs. measured by Vickers indenter tester). Pearson correlation and linear regression were performed to evaluate the correlation between filler weight and mechanical properties.

## 3. Results

### 3.1. Filler Content

Table 3 compares the filler weight provided by the manufacturer and the average filler weight obtained by the ash method in the current study. The inorganic filler weight of all the 3D-printed materials was approximately 33%, a filler percentage that is significantly lower than that of all CAD/CAM blocks.

### 3.2. Flexural Strength and Modulus

**Figure 2:**
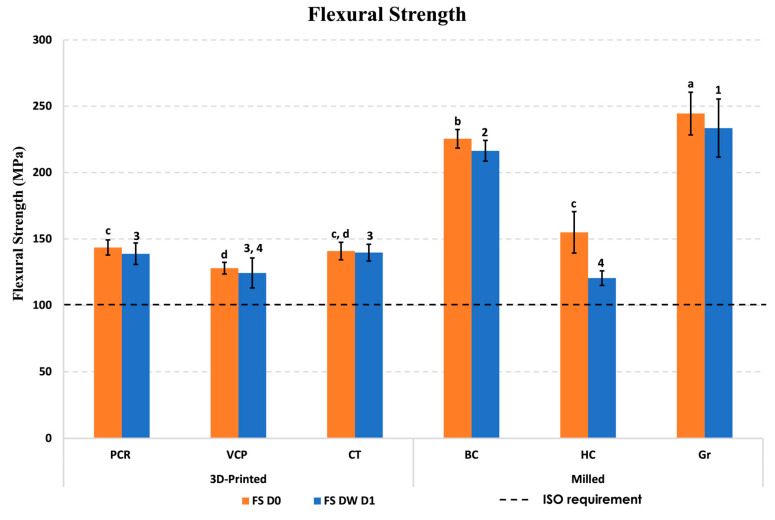
Flexural strength (FS) of the investigated materials at baseline (D0) and after 1-day storage in distilled water (DW D1). Different superscript letters indicate significant differences in FS between different materials at D0 (*p* ≤ 0.05), while different superscript numbers indicate significant differences in FS between different materials at DW D1 (*p* ≤ 0.05).

The flexural strength (FS) results of the investigated materials at baseline (D0) and after 1-day storage in distilled water (DW D1) are illustrated in Figure 2. At D0, FS of the 3D-printed materials, PCR, CT, and VCP, were 143.6, 140.9, and 128 MPa, respectively, and FS of the milled blocks for Gr, BC, and HC were 244.5, 225.5, and 155 MPa, respectively. After DW D1, there was a minimal and insignificant drop observed in the FS of PCR, VCP, CT, and BC; a significant, minor decrease was observed for Gr (*p* = 0.029), and a highly significant reduction for HC (*p* < 0.001).

**Figure 3:**
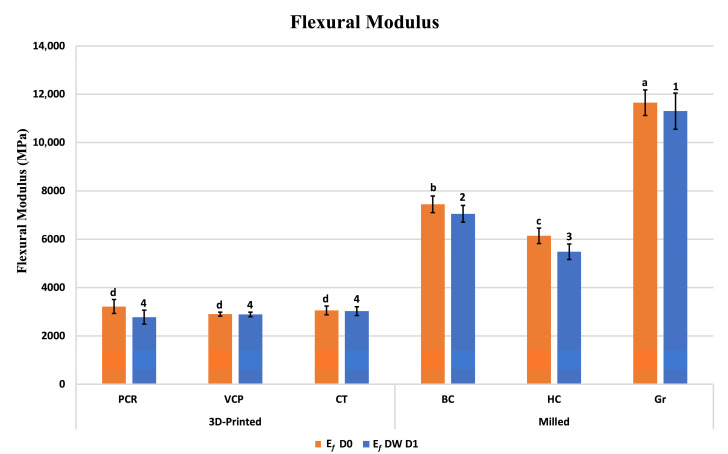
Flexural modulus (E*_f_*) of the investigated materials at baseline (D0) and after 1-day storage in distilled water (DW D1). Different superscript letters indicate significant differences in E*_f_* between different materials at D0 (*p* ≤ 0.05), while different superscript numbers indicate significant differences in E*_f_* between different materials at DW D1 (*p* ≤ 0.05).

Figure 3 depicts the flexural modulus (E*_f_*) of the investigated materials at baseline (D0) and after 1-day immersion in distilled water (DW D1). At D0, E*_f_* values of the 3D-printed materials (PCR, CT, and VCP) were measured as 3217, 3054, and 2901 MPa, respectively. E*_f_* of the milled blocks (Gr, BC, and HC) were measured as 11,650, 7446, and 6142 MPa, respectively. After DW D1, a slight and insignificant decline was noted in the E*_f_* of VCP and CT; however, a statistically significant reduction was detected in the case of PCR (*p* = 0.008), BC (*p* = 0.017), HC (*p* < 0.001), and Gr (*p* = 0.033).

Furthermore, the two-way ANOVA showed that material type had a highly significant effect on both FS and FM (*p* < 0.001) (Table 4).

### 3.3. Martens Hardness and Indentation Modulus

HM and E*_iT_* of the investigated materials are presented in Table 5. For both properties, there were statistically significant differences between 3D-printed materials and milled blocks (*p* < 0.001). Also, there were statistically significant differences in HM and E*_iT_* between different milled resin composite blocks (*p* < 0.001).

HM of the 3D-printed resin composites was approximately equal, around 217 N/mm^2^, and in contrast, the HM of the milled resin composite blocks exhibited a wider range from 434.7 to 858.4 N/mm^2^. Similarly, the E*_iT_* of the 3D-printed materials was relatively consistent, around 5.8 kN/mm^2^, while the E*_iT_* of the milled blocks demonstrated a diverse range, varying from 10.9 to 22.7 kN/mm^2^.

### 3.4. Vickers Hardness

Vickers hardness data of the investigated materials (measured by Zwick and measured by the Vickers indenter tester) are presented in Table 6. In both methods, there were statistically significant differences in HV between 3D-printed resin composites and milled resin composite blocks (*p* < 0.001). Also, there were statistically significant differences in HV measured by Zwick between different milled resin composite blocks (*p* < 0.001), and there were statistically significant differences in HV measured by the Vickers indenter tester between Gr and both BC and HC (*p* < 0.001). T-test indicates statistically significant differences in HV of VCP, BC, and HC materials measured by Zwick vs. those measured by the Vickers indenter tester (*p* < 0.001).

## 4. Discussion

The findings of the current study indicate that the tested materials were significantly different in their mechanical properties (flexural strength, Martens hardness, Vickers hardness, and flexural and elastic modulus). Also, the 3D-printed definitive resin composites were significantly different from the milled ones in terms of mechanical properties. Consequently, both null hypotheses were rejected.

### 4.1. Filler Content

The mechanical properties of resin composites are influenced by their composition [20], and filler content showed a strong association with the mechanical strength of materials [21]. In this study, milled resin composite blocks exhibited significantly higher filler content than 3D-printed composites. The relatively low filler content in 3D-printed resin composites can be attributed to the need for liquid consistency during 3D printing [22]. Moreover, this study observed that the milled composite blocks had varying filler weight percentages, while the 3D-printed composites had almost the same filler weight percentage. Consequently, there were notable differences in the tested mechanical properties between the investigated milled composite blocks and between the milled and 3D-printed composites, but not among the different 3D-printed composites.

**Figure 4:**
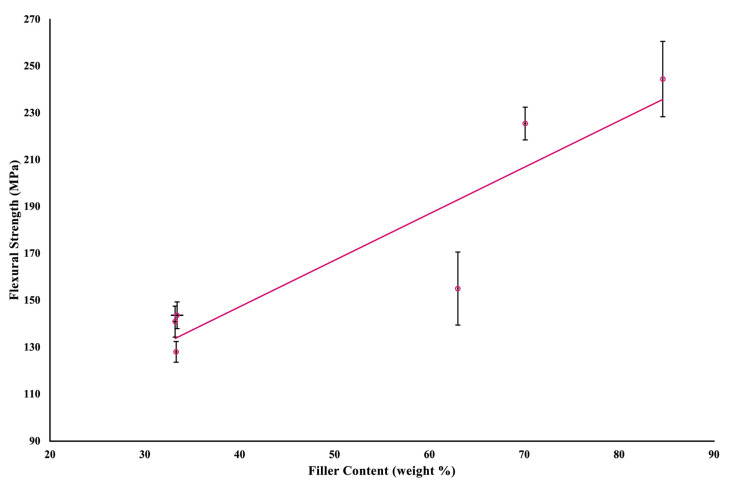
A scatter plot and linear regression of the correlation between flexural strength and filler weight. The circles represent the mean values, and the lines indicate the standard deviations.

**Figure 5:**
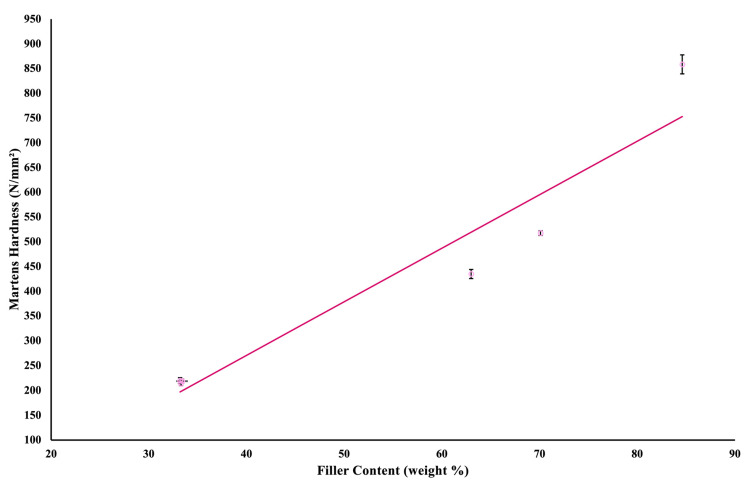
A scatter plot and linear regression of the correlation between Martens hardness and filler weight. The circles represent the mean values, and the lines indicate the standard deviations.

A linear regression (Figure 4) assessed the correlation between filler weight (%) and flexural strength of the investigated materials; the model was statistically significant, R^2^ = 0.832, F (1,4) = 19.86, *p* = 0.011, and adjusted R^2^ = 0.790. The presence of a sigmoidal pattern in the linear regression model could be attributed to the relatively small groups; a more reliable correlation would be possible by conducting the analysis using identical material with varying sets of filler weights. Figure 5 represents another statistically significant linear regression examining the correlation between the filler content and the Martens hardness of the investigated materials, R^2^ = 0.921, F (1,4) = 46.67, *p* = 0.002, and adjusted R^2^ = 0.901. Both linear regressions show that variations in the filler content of the investigated materials explain differences in their mechanical properties.

Despite this, the regression analyses were based on six materials only, which limits the interpretation of the findings and prevents them from being considered predictive. Rather, they provide a preliminary indication that filler weight may contribute to mechanical performance, which is consistent with previous findings that various parameters associated with fillers, including filler loading and its particle size, shape, and distribution, have been found to affect the physical and mechanical properties of dental resin composites [23,24,25].

### 4.2. Flexural Strength and Modulus

The results from D0 testing revealed that the flexural strength (FS) and flexural modulus (E*_f_*) of milled blocks were significantly higher than those of the 3D-printed resin composites. This finding is consistent with prior research [22], which demonstrated a significantly higher FS and E*_f_* in milled blocks (Gr and BC) than that of the tested 3D-printed material (VCP). According to the same study [22], the flexural strength was seen to follow a decreasing order, namely Gr > BC > VCP, which agrees with our findings.

FS of the Gr, BC, HC, and VCP at D0 conflict with other studies that reported lower FS values than those measured in this study for the same materials. FS were Gr 244.5 vs. 186.02 [22], BC 225.5 vs. 170.29 [22], HC 155 vs. 120.38 [26], and VCP 128 vs. 119.85 [22] MPa. After DW D1, the FS of the milled blocks Gr, BC, and HC (233.6, 216.5, and 120.5 MPa, respectively) were comparable with the result of a previous study (244.9, 213.3, and 132.3 MPa, respectively) [27] for the same tested blocks. FS of 3D-printed materials VCP and CT at DW D1 were higher than those reported in a previous study (124.4 vs. 101.8 [14] and 139.7 vs. 125.9 [14] MPa, respectively). The observed disparities could possibly be due to variations in the specimen dimensions across studies. Moreover, the observed decline in FS and E*_f_* at DW D1 in the present investigation can be ascribed to the alterations in the internal structure of the materials caused by water sorption [28].

Despite these variations, both the 3D-printed and milled materials showed clinically acceptable initial flexural strength, surpassing the ISO minimum requirement (100 MPa) for polymer-based restorative materials (specifically, type 1 class 2 group 2 restorative material). Nevertheless, these materials exhibit different degrees of reduction in flexural strength following water storage. Further studies are required in order to have a better understanding of the long-term performance of these dental materials in clinical scenarios.

### 4.3. Hardness

#### 4.3.1. Martens Hardness and Indentation Modulus

The Martens hardness (HM) and indentation modulus (E*_iT_*) measurements of the examined materials exhibit significant variations between the two manufacturing techniques and among the various resin composite blocks. The observed discrepancy in HM values between 3D-printed and milled materials indicates that the latter exhibit superior resistance to plastic deformation. The finding aligns with the existing literature, showing that milled restorations are associated with superior microhardness than their 3D-printed counterparts [29], which can be attributed to the dense and uniform structure achieved through milling techniques [30] compared with 3D-printed counterparts, which may possess inherent microstructural irregularities owing to the layer-by-layer additive manufacturing process [29].

#### 4.3.2. Vickers Hardness

The HV values found in this study for PCR, CT, HC, and Gr (29.5, 30.3, 67.2, and 119.1, respectively) were comparable to the values reported in previous studies (29.39 [31], 30.1 [32], and 70 and 120 [19], respectively). In contrast, the HV value of BC (68.6) was lower than a previously reported value (80) [19].

Furthermore, the present results reveal significant variations in HV measurements of VCP, BC, and HC materials when comparing the Martens indentation test to the Vickers indenter tester (*p* < 0.001). The observed disparities in the results could be ascribed to the divergent approaches employed by the two methods. The Vickers indenter tester is known to exhibit inherent subjective variation because the determination of the indentation surface area is based on the average length of both diagonals, which is subjectively determined through microscopic observation by the naked eye [33]. The accuracy of these measurements may be influenced by many factors, including the resolution of the optical system, the perception of the operator, and the elastic recovery of the material [11]. These variables contribute to the observed variability in results.

### 4.4. Flexural Modulus vs. Indentation Modulus

**Figure 6:**
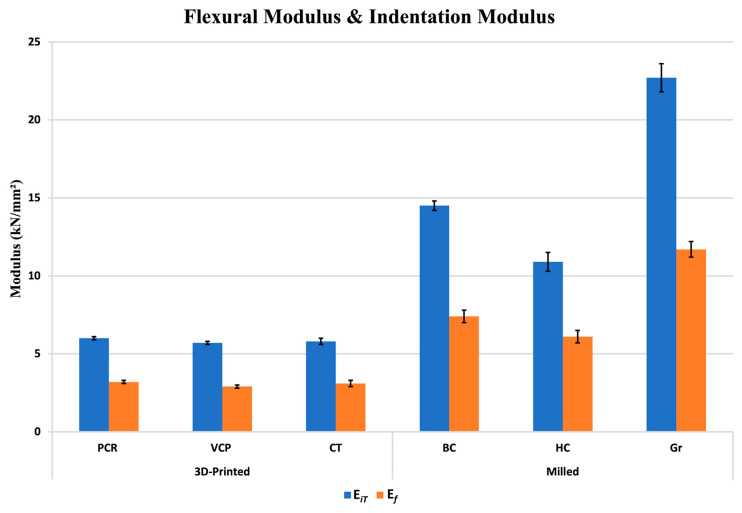
Flexural modulus and indentation modulus. *t*-test indicates a significant difference between the flexural modulus and the indentation modulus (*p* < 0.001).

To compare the results of the E*_f_* and E*_iT_*, the E*_f_* values were converted from MPa units to kN/mm^2^ units. Figure 6 compares the E*_f_* and E*_iT_* of the investigated materials in (kN/mm^2^). The *t*-test indicates a significant difference between the E*_f_* and E*_iT_* (*p* < 0.001). The dissimilarity between E*_f_* and E*_iT_* measurements in the tested materials can be clarified by their distinctive testing procedures. The E*_f_*, ascertained through conducting bending tests, provides an indicator of the ability of the material to withstand deformation when subjected to flexural stress [34]. On the other hand, E*_iT_* evaluates the capacity of a material to withstand localised deformation when subjected to compressive stresses [35]. The disparity in testing methodologies may account for the elevated values of E*_iT_* since materials tend to demonstrate stronger resistance to localised compressive pressures than bending stresses.

### 4.5. Methodological Considerations and Future Research

All 3D-printed specimens were built in a vertical orientation, which has been previously shown to result in 3D-printed resin composite specimens with superior mechanical properties [13,14]. Although this orientation was selected to standardise specimen fabrication, the inherent anisotropy of the 3D-printed resin composites was not fully assessed [36]. Future studies should evaluate multi-directional mechanical behaviour to further characterise anisotropic responses.

Moreover, VarseoSmile Crown Plus was manufactured by BEGO (Germany). Shortly thereafter, BEGO collaborated with Formlabs to allow for the processing of the materials using the Form 3B printer (Formlabs, Somerville, MA, USA) and introduced the material as Permanent Crown Resin. In this study, both materials were tested, yielding different results. These differences likely reflect variations in the 3D printing systems [37]. Stereolithography (SLA; Form 3B, Formlabs, Somerville, MA, USA) utilises a light source wavelength and curing procedure different from those of Digital Light Processing (DLP; Asiga MAX, Asiga, Sydney, Australia). Further investigations should examine the influence of 3D printer type and post-curing conditions, including post-curing device, temperature, and exposure time, on the mechanical properties. Additionally, future research should examine other properties, such as fracture-toughness and fractographic analysis, and incorporate extended ageing protocols, clinical evaluations of wear resistance, and durability to provide deeper insight into the long-term mechanical performance of these materials.

### 4.6. Clinical Implications

Despite their lower strength and hardness, 3D-printed resin composites may still be clinically acceptable for certain clinical scenarios, such as for single-unit restorations or in low-to-moderate-load regions. However, their long-term performance and behaviour under complex intraoral conditions remain insufficiently characterised. Therefore, careful case selection is essential when considering 3D-printed materials for definitive restorations.

## 5. Conclusions

Within the limitations of this study, the following conclusions can be drawn:

1. Filler content was strongly associated with flexural strength and Martens hardness of the tested materials.
2. Milled composites exhibited superior mechanical properties due to their higher filler content compared with 3D-printed materials.
3. All tested materials exceeded the ISO minimum requirements for flexural strength (100 MPa); however, the milled resin composites showed markedly higher stiffness and hardness.

## Figures and Tables

**Table 1:** Investigated materials and manufacturer information.

	Material Name	Composition	Inorganic Fillers	Manufacturer
**3D-printed**	Permanent Crown Resin (PCR)	Esterification products of 4,4’-isopropylidiphenol, ethoxylated and 2-methylprop-2enoic acid, ethoxylated bisphenol A dimethacrylate (Bis-EMA, methacrylate polymer) methyl benzoylformate, diphenyl (2,4,6-trimethylbenzoyl) phosphine oxide (TPO, photoinitiator)	Silanized dental glass	Formlabs (Somerville, MA, USA)
	VarseoSmile Crown plus (VCP)	Esterification product of 4.4‘-isopropylidiphenol, ethoxylated and 2-methyl- prop-2enoic acid. methyl benzoylformate, diphenyl (2,4,6-trimethylbenzoyl) phosphine oxide (TPO, photoinitiator)	Silanized dental glass	BEGO GmbH & Co. KG (Bremen, Germany)
	Crowntec (CT)	BisEMA, trimethylbenzoyldiphenylphosphine oxide (TPO, photoinitiator)	Pyrogenic silica Silanized dental glass	Saremco Dental AG (Rebstein, Switzerland)
**Milled**	Brilliant Crios (BC)	Cross-linked methacrylates (Bis-GMA, Bis-EMA, TEGDMA)	Barium glass and amorphous silica	COLTENE (Altstatten, Switzerland)
	Shofu Block HC (HC)	UDMA, TEGDMA	Silica powder, microfumed silica, and zirconium silicate	Shofu (Kyoto, Japan)
	Grandio Blocs—HT (Gr)	14% UDMA, DMA	Nanohybrid fillers	VOCO GmbH (Cuxhaven, Germany)

**Table 2:** 3D-printed resin composites printing, washing, and post-curing process.

Material Name	3D Printer/Techniques	Cleaning Solution in Form Wash	Post-Curing	Post-Curing Device
PCR	Form 3B (Formlabs)/(SLA)	99% isopropyl alcohol	2 × 20 min at 60 °C	Form Cure (Formlabs, Somerville, MA, USA)
VCP	Asiga MAX/(DLP)	96% ethanol	2 × 1500 flashes	Otoflash (BEGO GmbH & Co. KG, Bremen, Germany)
CT	Asiga MAX/(DLP)	99% isopropyl alcohol	2 × 2000 flashes	Otoflash (BEGO GmbH & Co. KG, Bremen, Germany)

**Table 3:** Filler content of the investigated materials.

Material Name	Manufacturer Filler Weight %	Measured Filler Weight % Mean (SD)
PCR	30–50	33.4 (0.6)
VCP	30–50	33.3 (0.05)
CT	30–50	33.2 (0.09)
BC	70	70.1 (0.05) [19]
HC	61	63.0 (0.02) [19]
Gr	86	84.6 (0.01) [19]

**Table 4:** Results of two-way ANOVA for the effects of material type, storage condition, and their interaction on flexural strength (FS) and flexural modulus (FM).

Parameter	Source of Variation	Sum of Squares	df	F	*p* Value	Effect Size
FS	Material	240,013.479	5	396.567	<0.001	0.948
	Storage	3407.247	1	28.148	<0.001	0.207
	Material × Storage	3735.946	5	6.173	<0.001	0.222
FM	Material	1,158,706,936.666	5	1767.244	<0.001	0.988
	Storage	2,964,163.333	1	22.605	<0.001	0.173
	Material × Storage	1,569,736.667	5	2.394	=0.042	0.100

**Table 5:** Martens hardness and indentation modulus of the investigated materials.

Material Name	Martens Hardness (N/mm^2^) Mean (SD)	Indentation Modulus (kN/mm^2^) Mean (SD)
PCR	218.6 ^d^ (4.2)	6.0 ^d^ (0.1)
VCP	213.6 ^d^ (4)	5.7 ^d^ (0.1)
CT	219.7 ^d^ (5.9)	5.8 ^d^ (0.2)
BC	517.3 ^b^ (4.6)	14.5 ^b^ (0.3)
HC	434.7 ^c^ (9.2)	10.9 ^c^ (0.6)
Gr	858.4 ^a^ (19.2)	22.7 ^a^ (0.9)

Within each column, a different superscript letter indicates a significant difference in the measured property between different materials (*p* ≤ 0.05).

**Table 6:** Vickers hardness of the investigated materials (measured by Zwick and Vickers indenter tester).

Material Name	Measured by Zwick (HV) Mean (SD)	Measured by Vickers (HV) Mean (SD)
PCR	29.8 ^d^ (0.6)	29.5 ^c^ (0.8)
VCP	29.3 ^d^ (0.7)	31.2 ^c^ (0.6)
CT	30.3 ^d^ (0.8)	30.3 ^c^ (1)
BC	70.2 ^b^ (0.7)	68.6 ^b^ (0.9)
HC	62.1 ^c^ (1.7)	67.2 ^b^ (3)
Gr	119.5 ^a^ (2.3)	119.1 ^a^ (3.2)

Within each column, a different superscript letter indicates a significant difference in the measured property between different materials (*p* ≤ 0.05).

## Data Availability

The original contributions presented in the study are included in the article, and further inquiries can be directed to the corresponding author.
